# Association of plasma angiogenin with risk of major cardiovascular events in type 2 diabetes

**DOI:** 10.1186/s12933-024-02156-8

**Published:** 2024-02-15

**Authors:** Resham L. Gurung, Sylvia Liu, Jian-Jun Liu, Yiamunaa M., Huili Zheng, Clara Chan, Keven Ang, Tavintharan Subramaniam, Chee Fang Sum, Su Chi Lim

**Affiliations:** 1 Clinical Research Unit, Khoo Teck Puat Hospital, Singapore, Singapore; 2 Diabetes Centre, Admiralty Medical Centre, Singapore, Singapore; 3 Saw Swee Hock School of Public Heath, Singapore, Singapore; 4 Lee Kong Chian School of Medicine, Nanyang Technological University, Singapore, Singapore; 5 Cardiovascular and Metabolic Disorders Signature Research Program, Duke-NUS Medical School, Singapore, Singapore

**Keywords:** Angiogenin, Major adverse cardiovascular events, Type 2 diabetes

## Abstract

**Background:**

Angiogenin, an enzyme belonging to the ribonucleases A superfamily, plays an important role in vascular biology. Here, we sought to study the association of plasma angiogenin and major adverse cardiovascular events (MACEs) in patients with type 2 diabetes (T2D).

**Methods:**

This prospective study included 1083 T2D individuals recruited from a secondary hospital and a primary care facility. The primary outcome was a composite of four-point MACE (nonfatal myocardial infarction, stroke, unstable angina pectoris leading to hospitalization and cardiovascular death). Circulating angiogenin was measured by a proximity extension assay. Cox regression models were used to evaluate the association of baseline plasma angiogenin with the risk of MACE.

**Results:**

During a median follow-up of 9.3 years, 109 (10%) MACE were identified. Plasma angiogenin was significantly higher in participants with MACE than in those without MACE (*P* < 0.001). Doubling of plasma angiogenin concentration was associated with a 3.10-fold (95% CI 1.84–5.22) increased risk for MACE. The association was only moderately attenuated after adjustment for demographic and cardiometabolic risk factors (adjusted HR 2.38, 95% CI 1.34–4.23) and remained statistically significant after additional adjustment for estimated glomerular filtration rate (eGFR) and urinary albumin to creatinine ratio (uACR) (adjusted HR 1.90, 95% CI 1.02–3.53). A consistent outcome was obtained when plasma angiogenin was analysed as a categorical variable in tertiles.

**Conclusions:**

Plasma angiogenin was associated with the risk of future cardiovascular events in patients with T2D and may be a promising novel biomarker for identifying high-risk T2D patients for early management.

**Supplementary Information:**

The online version contains supplementary material available at 10.1186/s12933-024-02156-8.

## Background

Patients with type 2 diabetes (T2D) are at increased risk of major adverse cardiovascular events (MACEs), including myocardial infarction and stroke compared to those without diabetes [1, 2]. Clinical studies and animal model suggest that diabetes accelerates the progression of atherosclerotic lesions and may also lead to defects in the remodelling of plaques after cholesterol reduction [3]. In patients with T2D, non-glycaemic risk factors were also associated strongly with cardiovascular disease (CVD) [4]. Mounting evidence supports that hypertension, obesity, dyslipidaemia and impaired kidney function are major risk factors underlying the increased risk of CVD among patients with diabetes. In this regard, European Heart Association recently developed the SCORE2-Diabetes score for the stratification of CVD risk in patients with diabetes [5]. However, traditional risk factors, such as age, blood pressure, body mass index, and lipid profile, may be insufficient in risk- prediction. With the rising prevalence of T2D [6], identifying novel biomarkers for CVD is paramount to aid efforts for early intervention and management of high-risk T2D patients. On the other hand, novel biomarkers may shed light on the pathophysiology of CVD and guide the selection of new targets for drug development.

Plasma angiogenin, a 14 kDa molecular weight extracellular protein and a member of the human ribonucleases A superfamily of enzymes, is a potent angiogenic factor [7]. Angiogenin, first identified as an oncogenic protein, induces blood vessel formation in vitro and in vivo to promote angiogenesis [8]. Several reports have highlighted the involvement of angiogenin in neuroprotection [9], inflammation [10], and regeneration of damaged tissues [11]. Angiogenin also plays a role in endothelial cell survival, proliferation and migration, which are crucial in vascular homeostasis [11].

Emerging evidence suggests that plasma angiogenin is significantly higher in CVD [12]. Plasma angiogenin levels were elevated in patients with coronary artery disease [13], acute coronary syndrome (ACS) [14] and heart failure with preserved ejection fraction [15]. In addition, higher angiogenin levels were also predictive of adverse events (composite of cardiovascular death, recurrent ACS, revascularization and heart failure) in the short term (6 months) in patients with ACS [14]. However, these studies were performed in a small number of patients. Moreover, there is a paucity of data on the role of angiogenin in incident cardiovascular events in T2D patients. We hypothesized that plasma angiogenin may be associated with future cardiovascular events in T2D patients. Therefore, we first evaluated the association between plasma angiogenin and the incidence of MACEs in multiethnic Asians with T2D. We also explored whether plasma angiogenin might improve the prediction ability of MACE above the European Heart Association SCORE2-Diabetes.

## Methods

### Study population

This study included T2D participants in the longitudinal Singapore Study of Macroangiopathy and Microvascular Reactivity in Type 2 Diabetes (SMART2D) cohort [16]. A total of 2057 outpatients with T2D were recruited consecutively from a secondary hospital and a primary care medical facility in Singapore between August 2011 and March 2014. T2D was diagnosed by attending physicians according to prevailing American Diabetes Association (ADA) criteria after exclusion of type 1 diabetes mellitus (T1DM) and diabetes attributable to specific causes [17]. Individual with diabetes with pregnancy, manifest infection, autoimmune disease, and cancer on active treatments were excluded from cohort enrolment as described previously [18, 19].

For the current study, only participants with baseline plasma angiogenin data available were included (*N* = 1171). Given that chronic kidney disease (CKD) is an established risk factor for CVD in patients with diabetes [20, 21], we excluded 88 patients with baseline eGFR less than 60 ml/min/1.73m^2^ to partly mitigate the confounding of CKD on the association between angiogenin and CVD (Supplementary Figure S1). Written consent was obtained from each participant. The study was approved by the Singapore National Healthcare Group Domain Specific Review Board.

### Participant follow-up and clinical outcomes

Participants were followed up by linkage with a centralized data repository, which collected hospitalization summaries, surgical procedures, outpatient visit records, imaging examinations and all biochemical assays. Participants were also invited for research visits in the clinical research unit in the hospital every three years, during which biosamples were obtained and major clinical outcomes during the past 3 years were queried and validated by reviewing medical records afterwards. We combined data from routine clinical care and research visits into one dataset to ascertain the outcome. Additionally, we queried the national death registry and obtained the primary cause of death on the death certificate. The primary outcome was a composite of nonfatal myocardial infarction, stroke, and unstable angina pectoris leading to hospitalization and cardiovascular death, whichever occurred first. Nonfatal AMI, stroke and unstable angina pectoris leading to hospitalization were identified according to the primary diagnosis on hospitalization discharge summary. Cardiovascular death was identified by the primary cause of death on the death certificate and cross- validated by data linkage with national death registry. As usually adopted by large clinical trials, we also combined these four major adverse cardiovascular events into a composite outcome (4-point MACE) to improve statistical power. In sensitivity analysis, we analysed the association of plasma angiogenin and each component of the 4-point MACE individually. This study was censored on 31 December 2021.

### Plasma angiogenin measurement

Baseline plasma angiogenin was measured on the Proximity Extension Assay–Based Proteomics Platform (O-link). Briefly, all samples were processed, and quality control was done by Olink [22]. The final assay readout is expressed in normalized protein expression (NPX) values, an arbitrary unit on a log2 scale with high values corresponding to high protein expression. The intra-assay coefficient of variance ranged between 7% and 8%, while the interassay coefficient of variance ranged between 11% and 14%.

### Covariates

Ethnicity, smoking status, and diabetes duration were self-reported. Information on medication usage at baseline was extracted from the medication dispensary database. History of atherosclerotic cardiovascular disease, including nonfatal myocardial infarction and stroke, was self-reported and cross-validated by reviewing medical records after cohort enrolment. HbA1c was measured by a point-of-care immunoassay analyser (DCA Vantage Analyser; Siemens, Munchen, Germany). Mean arterial pressure was calculated as.

$$\frac{\left[\text{systolic\,blood\,pressure} + (2 \times \text{diastolic\,blood pressure})\right]}{3}$$

Serum triacylglycerol, high-density lipoprotein (HDL) cholesterol, and low-density lipoprotein (LDL) cholesterol were measured by enzymatic methods (Roche Cobas Integra 700; Roche Diagnostics, Basel, Switzerland). Creatinine was quantified by an enzymatic method, which was traceable to the isotope dilution mass spectrometry reference. All eGFR readings in the current studies were estimated based on serum creatinine by the 2009 Chronic Kidney Disease-Epidemiology Collaboration formula [23]. Urine albumin was quantified by a solid-phase competitive chemiluminescent immunoassay (Immulite; DPC, Gwynedd, UK), and the albuminuria level was presented as the albumin-to-creatinine ratio (ACR, mg/g).

### Statistical analysis

Baseline continuous variables with a normal distribution are expressed as the mean ± standard deviation (SD), while nonnormally distributed variables are presented as medians (interquartile range, IQR). Categorical data were expressed as proportions. Comparison of baseline variables between those with and without MACE (control) was performed by independent sample t-test for normally distributed variables or the Mann–Whitney U test for non-normally distributed variables. Differences among plasma angiogenin tertiles were compared by one-way ANOVA, Kruskal-Wallis rank test or X^2^ test where appropriate. Urine ACR was log-transformed due to skewed distribution. The missing value in each clinical and biochemical variable was < 0.5% and handled by listwise deletion.

The cumulative incidence of MACE was visualized by the Kaplan–Meier method after stratifying participants by angiogenin tertiles. Differences in MACE incidence across tertiles were compared by the log-rank test. For the primary analysis, cox proportional hazard regression models were used to evaluate the association of plasma angiogenin (as continuous variables and tertiles) with MACE. Covariates were a priori selected by biological plausibility. Model 1 adjusted for age, sex (female as reference), ethnicity (Chinese as reference), CVD history (no as reference), smoking status (current versus others), body mass index, diabetes duration, HbA1c, mean arterial pressure, HDL-cholesterol, LDL-cholesterol, log-transformed triacylglycerol, statin, and aspirin usage (no as reference), Model 2 additionally adjusted for baseline eGFR and log-transformed urine ACR above model 1. Male participants had a higher level of angiogenin than female (Table 2). We therefore added the multiplicative term sex-by-angiogenin as a covariate in the model to assess whether sex modulated the association between angiogenin and MACE. The proportional hazard assumption was examined using Schoenfeld residuals, and no violation of proportionality was identified.

In an exploratory analysis, we evaluated whether angiogenin might improve the prediction ability of MACE above the European Heart Association SCORE2-Diabetes, a new algorithm for predicting the 10-year risk of CVD in individuals with T2D which included age, sex, smoking, SBP, total and HDL cholesterol, age at T2D diagnosis, HbA1c and eGFR [5]. Discrimination was evaluated by the area under the receiver-operating characteristic curve (AUC). Improvements in risk prediction were also quantified by median improvement in AUC from 1000 resamplings. Statistical analysis was performed using R-statistic programming (version 4.0.3, R Foundation for Statistical Computing, Vienna, Austria), Stata version 17 (StataCorp LP, College Station, TX, USA) and SPSS Version 27 (IBM Corp., Armonk, NY). A two-sided P value < 0.05 was considered statistically significant.

## Results

### Participant characteristics

A total of 1083 T2D patients with preserved kidney filtration function were included in the analysis (Supplementary Figure S1). Participants included in the current analysis had a mean ± SD age of 54 ± 11 years old, 50% were male, 49% were Chinese, 23% were Malay and 28% were Asian Indians. Compared to participants excluded in this study (*N* = 974), participants included were younger, had shorter diabetes duration, higher eGFR, lower urine ACR and were less likely to have atherosclerotic cardiovascular disease (ASCVD) history (Supplementary Table S1).

Over a median of 9.3 (IQR 8.4–9.8) years of follow-up (9,513 patient-years), 109 (10%) MACE events (58 nonfatal acute myocardial infarction [AMI], 27 nonfatal stroke, 21 CVD death and 28 unstable angina pectoris leading to hospitalization) were identified. The crude incidence of MACE was 1.15 per 100 patient-years. Participants who developed MACE were older, had a longer diabetes duration, higher HbA1c, higher blood pressure and poorer kidney function compared to those without incident MACE. In addition, they were more likely to be male, active smokers, have a history of ASCVD and on aspirin and statin treatments (Table 1). Of note, plasma angiogenin was significantly higher in patients who developed MACE compared to those without MACE occurrence.

**Table 1:** Baseline clinical and biochemical characteristics in participants stratified by MACE outcome

	With MACE (*N* = 109)	Without MACE (*N* = 974)	P value
Plasma angiogenin (NPX, IQR)	**1.12 (0.91–1.41)**	**0.95 (0.76–1.19)**	**< 0.001**
Index age (years)	**56.4 ± 9.2**	**53.2 ± 10.7**	**0.001**
Male sex (%)	**58.7**	**48.6**	**0.044**
Ethnicity (%)			**0.003**
Chinese	**35.8**	**50.9**	
Malay	**33.9**	**21.5**	
Asian Indian	**30.3**	**27.6**	
Diabetes duration (years, IQR)	**7 (4–15)**	**5 (3–10)**	**0.001**
Active smoker (%)	**15.7**	**9.2**	**0.032**
ASCVD history (%)	**16.5**	**5.0**	**< 0.001**
Body mass index (kg/m^2^)	28.7 ± 5.0	28.2 ± 5.4	0.387
HbA1c (%)	**8.1 ± 1.4**	**7.7 ± 1.3**	**0.005**
Blood pressure (mmHg)			
Systolic	**140 ± 18**	**137 ± 17**	**0.047**
Diastolic	**82 ± 9**	**80 ± 9**	**0.013**
Mean arterial pressure	**101 ± 10**	**99 ± 11**	**0.010**
Lipid profile (mM)			
HDL cholesterol	1.24 ± 0.30	1.29 ± 0.36	0.107
LDL cholesterol	2.92 ± 0.99	2.79 ± 0.80	0.189
Triacylglycerol (IQR)	1.40 (1.06–1.94)	1.35 (1.02–1.88)	0.592
Baseline renal function			
eGFR (ml/min/1.73m^2^)	**91 ± 16**	**99 ± 17**	**< 0.001**
urine ACR (µg/mg, IQR)	**26 (9-120)**	**15 (4–47)**	**< 0.001**
High sensitivity CRP (pg/ml)	2657 (666–5879)	2213 (762–4974)	0.282
Medication usage (%)			
Aspirin	**30.6**	**16.2**	**< 0.001**
Statin	**86.1**	**77.0**	**0.031**

Data were presented as mean ± SD, median (interquartile range, IQR) or percentages. Between group differences were compared by student *t* test, Mann-Whitney U test or X^2^ test where appropriate. ASCVD, atherosclerotic cardiovascular disease; eGFR, estimated glomerular filtration function; ACR, albumin-to-creatinine ratio; CRP, c-reactive protein. Variables differed significantly between groups have been highlighted in bold font

Compared to participants in the lower tertile of plasma angiogenin, those in higher tertiles had a lower level of HDL cholesterol, higher triacylglycerol, and poorer kidney function (a lower eGFR and higher uACR), and were more likely to be male, be active smokers, and have an ASCVD history (Table 2). Plasma angiogenin significantly correlated positively with diabetes duration, mean arterial pressure, triacylglycerol, urine ACR, high sensitivity c-reactive proteins (hs-CRP) and inversely with HDL-cholesterol and eGFR (Supplementary Table S2).

**Table 2:** Baseline clinical and biochemical characteristics in participants stratified by plasma angiogenin tertiles

	All participants (*N* = 1083)	Tertile 1 (*N* = 361)	Tertile 2 (*N* = 361)	Tertile 3 (*N* = 361)	P value
Plasma angiogenin (NPX, IQR)	**0.98 (0.77–1.22)**	**0.69 (0.61–0.77)**	**0.98 (0.91–1.04)**	**1.35 (1.22–1.55)**	**< 0.001**
Index age (years)	53.5 ± 10.6	53.7 ± 10.3	53.2 ± 10.6	53.6 ± 10.9	0.842
Male sex (%)	**49.6**	**34.9**	**52.6**	**61.2**	**< 0.001**
Ethnicity (%)					0.838
Chinese	49.4	49.3	48.2	50.7	
Malay	22.7	21.3	23.5	23.3	
Asian Indian	27.9	29.4	28.3	26.0	
Diabetes duration (years, IQR)	5 (3–12)	5 (3–10)	5 (3–11)	5 (3–13)	0.278
Active smoker (%)	**9.9**	**5.5**	**8.6**	**15.6**	**< 0.001**
ASCVD history (%)	**6.2**	**4.4**	**4.4**	**9.7**	**0.003**
Body mass index (kg/m^2^)	28.3 ± 5.4	28.1 ± 5.4	28.5 ± 5.8	28.2 ± 4.9	0.493
HbA1c (%)	7.7 ± 1.3	7.7 ± 1.2	7.7 ± 1.3	7.8 ± 1.4	0.376
Blood pressure (mmHg)					
Systolic	137 ± 17	135 ± 17	138 ± 18	138 ± 17	0.050
Diastolic	**80 ± 9**	**79 ± 9**	**80 ± 9**	**80 ± 9**	**0.011**
Mean arterial pressure	**99 ± 11**	**98 ± 11**	**99 ± 11**	**100 ± 10**	**0.008**
Lipid profile (mM)					
HDL cholesterol	**1.29 ± 0.36**	**1.35 ± 0.34**	**1.30 ± 0.38**	**1.22 ± 0.33**	**< 0.001**
LDL cholesterol	2.81 ± 0.82	2.74 ± 0.78	2.86 ± 0.84	2.82 ± 0.84	0.115
Triacylglycerol (IQR)	**1.35 (1.02–1.88)**	**1.26 (0.92–1.72)**	**1.36 (1.05–1.87)**	**1.46 (1.08–2.17)**	**< 0.001**
Baseline renal function					
eGFR (ml/min/1.73m^2^)	**98 ± 17**	**103 ± 15**	**98 ± 17**	**92 ± 17**	**< 0.001**
Urine ACR (µg/mg, IQR)	**15 (5–49)**	**10 (3–32)**	**14 (5–40)**	**26 (8-104)**	**< 0.001**
Hs-CRP(pg/ml, IQR)	2279 (761–8013)	2078 (678–4819)	2323 (735–4977)	2504 (852–5365)	0.209
Medication usage (%)					
Aspirin	**17.6**	**11.7**	**17.2**	**24.2**	**< 0.001**
Statin	77.9	75.3	78.5	80.0	0.296

Data were presented as mean ± SD, median (interquartile range, IQR) or percentages. Among group differences were compared by one-way ANOVA, Kruskal-Wallis rank or X^2^ test where appropriate. ASCVD, atherosclerotic cardiovascular disease; eGFR, estimated glomerular filtration function; ACR, albumin-to-creatinine ratio; hs-CRP, high sensitivity C-reactive protein. Variables differed significantly among groups have been highlighted in bold font

Male participants had a higher level of plasma angiogenin than their female counterparts (median 1.04 [IQR 0.85–1.30] vs. 0.90 [IQR 0.70–1.14] per increment in NPX, *P* < 0.001)(results not shown). However, angiogenin did not interact with sex in association with MACE in the unadjusted analysis (*P* = 0.53 for interaction term). Therefore, we combined male and female participants in the subsequent analysis.

### Association of plasma angiogenin with incident MACE

**Fig. 1:**
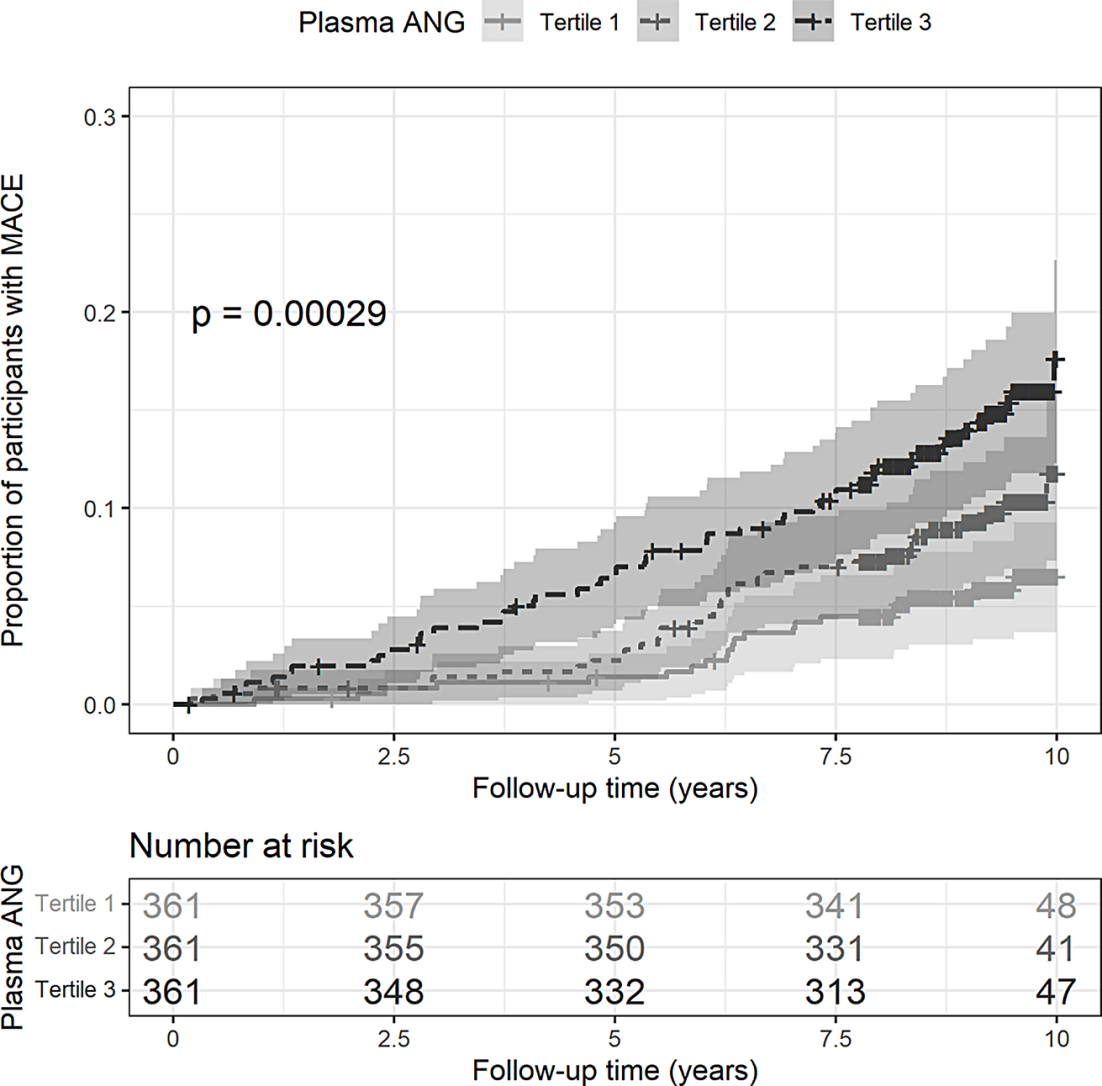
Cumulative Risk for MACE stratified by plasma angiogenin tertiles

Participants with plasma angiogenin in the higher tertile had a higher cumulative incidence of MACEs than those in the lowest tertile (*P* < 0.001) (Fig. 1). The cause-specific Cox regression model showed that one NPX increment i.e., doubling of plasma angiogenin concentration was associated with a 3.10-fold (95% CI 1.84–5.22, per increment NPX) increased risk for MACE (Unadjusted model, Table 3). Plasma angiogenin was associated with an increased risk of MACE, independent of demographic and cardiometabolic risk factors (adjusted HR 2.38, 95% CI 1.34–4.23, model 1) and remained statistically significant after further adjustment for eGFR and uACR, two strong risk factors for MACE among individuals with T2D (adjusted HR 1.90, 95% CI 1.02–3.53, model 2). A consistent outcome was obtained when plasma angiogenin was analysed as a categorical variable in tertiles (Table 3).

**Table 3:** Association of plasma angiogenin with MACE

Plasma angiogenin	Unadjusted Model		Multivariable Model 1		Multivariable Model 2	
	HR (95% CI)	P value	HR (95% CI)	P value	HR (95% CI)	P value
Continuous variable (per 1 unit increment)*	3.10 (1.84–5.22)	< 0.001	2.38 (1.34–4.23)	0.003	1.90 (1.02–3.53)	0.044
**Categorical variable**						
Lower tertile	Reference		Reference		Reference	
Intermediate tertile	1.67 (0.96–2.92)	0.069	1.46 (0.83–2.58)	0.187	1.33 (0.75–2.36)	0.330
Upper tertile	**2.72 (1.62–4.56)**	**< 0.001**	**1.98 (1.15–3.42)**	**0.014**	1.63 (0.91–2.89)	0.098

Cox proportional hazard regression model: time to 4-point MACE as outcome

Model 1 adjusted for age, sex, ethnicity, CVD history (yes or no), smoking status (current versus others), body mass index, diabetes duration, HbA1c, mean arterial pressure, HDL-cholesterol, LDL-cholesterol, log-transformed triacylglycerol, statin and aspirin usage

Model 2 additionally adjusted for baseline eGFR and log-transformed urine ACR above model 1

* One NPX increment is interpreted as doubling of plasma angiogenin concentration measured by proximity extension assay

### Prediction of incidence of MACE by plasma angiogenin above traditional risk factors

Adding plasma angiogenin to predictors in the SCORE2-Diabetes score did not significantly improve the C-index (AUC 0.698 to 0.701, delta 0.003, 95% CI -0.015-0.022).

### Sensitivity analysis

Additional adjustments for hs-CRP, did not materially alter the association between plasma angiogenin and risk of MACE (adjusted HR 1.86 [95% CI 0.99–3.48], per increment NPX) (data not shown). Plasma angiogenin was significantly associated with incident AMI in both univariable (HR 4.02 [95% CI 2.00-8.09]) and multivariable analyses (adjusted HR 2.87 [95% CI 1.25–6.58]) (Supplementary Table S3). It was also significantly associated with nonfatal stroke in univariate model but the association did not reach statistical significance in multivariable analysis (adjusted HR 2.82 [95% CI 0.85–9.37]) (Supplementary Table S3). Plasma angiogenin was not associated with cardiovascular death and unstable angina pectoris hospitalisation or all-cause mortality (Supplementary Tables S1, S4).

Notably, plasma angiogenin was associated with 2.35-fold risk of MACE (multivariable adjusted ,95% CI 1.01–5.43) in T2D patients with normal kidney function (eGFR > 90 ml/min/1.73m^2^) at baseline (Supplementary Table S5). The association of angiogenin with MACE was generally consistent in subgroup analyses after stratifying participants by ASCVD history, albuminuria category (ACR < 30 versus ≥ 30 mg/g) and HbA1c level (< 8% versus ≥ 8%) (Supplementary Table S6).

## Discussion

In this prospective study, we found that an increased plasma angiogenin level was associated with an increased risk of incident MACE, independent of cardiometabolic risk factors, including renal function, in T2D patients with preserved kidney function. Our findings suggest that angiogenin may play a role in the pathogenesis of MACE and holds promise as a novel biomarker for the early identification of patients with T2D at higher risk of CVD.

To our knowledge, this may be the first study to demonstrate the association of plasma angiogenin with the development of CVD in T2D patients. Previously, it was reported that plasma angiogenin levels are higher in patients with chronic heart failure [24, 25] and coronary artery disease than in healthy controls in cross-sectional studies. Tello-Montoliu and colleagues also showed that in patients (*N* = 440) with acute coronary syndrome (ACS), a high level of angiogenin is associated with adverse events at the six-month follow-up [14]. However, these studies were conducted in smaller cohorts and individuals without diabetes, with different disease settings and shorter follow-up periods. A recent meta-analysis reported by Yu et al. demonstrated that the plasma angiogenin levels does not differ significantly between T2D patients and those without diabetes [12]. However, it is important to note that the total sample size in the meta-analysis for each study was small and the heterogeneity among studies were significant. In our study, we only observed a modest positive correlation between plasma angiogenin levels and diabetes duration, mean arterial pressure, triglyceride and an inverse correlation with HDL-cholesterol and kidney function. Importantly, plasma angiogenin was associated with the risk of MACE independent of these clinical risk factors. Therefore, this finding suggests plasma angiogenin may modulate the risk of MACE in novel pathways and warrants further studies to elucidate the pathophysiologic role in the pathogenesis of CVD in the diabetic population.

CKD is an established risk factor for CVD in patients with diabetes [20, 21], and a cross-sectional study among 108 participants revealed that plasma angiogenin increases with advanced CKD stages [26]. Our findings highlight that the association between plasma angiogenin with risk of MACE is independent of clinical risk factors including kidney impairment. We observed that the association between plasma angiogenin, and MACE was only moderately modified by kidney function. First, the study was conducted in patients with preserved eGFR (above 60 ml/min/1.73 m^2^). The strength of the correlation between angiogenin and eGFR or ACR was moderate (Spearman rho < 0.3). This may differ from the early study by Choi et al., conducted in patients with advanced CKD and showed a strong correlation between angiogenin and eGFR. Second, the point estimate of HR was only moderately attenuated after adjustment for renal impairment biomarkers eGFR and ACR (multivariable models 1 and 2, Table 3).

Angiogenin, one of the strongest angiogenic factors, interacts with endothelial cells and initiates angiogenesis [11]. It has also been suggested that angiogenin is needed for vascular endothelial growth factor (VEGF) to stimulate angiogenesis, interacts with proteases that activate wound healing, such as the metalloproteinase family, and stimulates tissue plasminogen activator (tPA) to produce plasmin [27, 28] and is associated with the destabilization of atherosclerotic plaques. Hence, it is postulated that high levels of plasma angiogenin may be a biomarker of unstable plaque. Inflammation is an established risk factor for CVD [29] and plasma angiogenin levels are increased in response to inflammation [30]. However, the association observed between plasma angiogenin, and the incidence of MACEs was independent of CRP, suggesting the involvement of noninflammatory processes. Experimental models revealed that hypoxia-inducible factor (HIF) increases the angiogenic expression under conditions of low oxygen availability [31, 32] while treatment with the sodium-glucose cotransporter 2 inhibitor canagliflozin decreases the production of angiogenin [33]. While our study provides a plausible link between plasma angiogenin and CVD pathology, further studies are warranted to elucidate the exact mechanism.

The strength of the present analysis includes relatively large study population, in multiethnic Asians, with a long-term follow-up. However, we acknowledge the limitations of the study. First, due to the observational nature of the study, we are unable to infer causality between plasma angiogenin and cardiovascular events. Second, we were unable to determine the tissue and cellular source of plasma angiogenin [34]. Hence, future studies are necessary to elucidate the molecular pathways through which angiogenin exerts its effects on CVD development. Third, plasma angiogenin was measured by a semiquantitative approach. However, we observed that plasma angiogenin measured by PEA on O-link platform and that by conventional immunosorbent technology from an independent source are comparable and highly correlated (Spearman’s correlation coefficient 0.85) (Supplementary Figure S2). Next, due to the small event numbers, the statistical power for association between plasma angiogenin (categorical analysis) may be lower, resulting in broader confidence interval. In addition, we are unable to comprehensively evaluate the association of plasma angiogenin with individual components of MACE. Last, we do not have an external cohort to validate our findings. Hence, we are unsure whether our findings from this Southeast Asia cohort may be generalizable to other ethnic groups. In addition, our analysis was conducted in patients with preserved eGFR. Future studies are also warranted to examine the relationship between plasma angiogenin and diabetic patients with a broad spectrum of glomerular filtration function.

## Conclusion

A high level of plasma angiogenin is associated with an increased risk of cardiovascular events independent of traditional risk factors in patients with type 2 diabetes and preserved kidney filtration function, suggesting that plasma angiogenin may play a significant role in the pathogenesis of CVD. Our finding may pave the avenue for future preclinical and clinical studies to further characterize the role of angiogenin in the pathological pathway leading to cardiovascular disease in individuals with diabetes.

### Electronic supplementary material

Below is the link to the electronic supplementary material.

**Supplementary Material 1**: Supplementary Figures and Tables

## Data Availability

The datasets generated during and/or analyzed during the current study are available from the corresponding author on reasonable request.
