# Supplementary material for: Association of plasma angiogenin with risk of major cardiovascular events in type 2 diabetes
Source: Cardiovasc Diabetol. 2024 Feb 15;23:70. doi: 10.1186/s12933-024-02156-8 (PMC10870605; doi:10.1186/s12933-024-02156-8)
Supplement: Supplementary file 1 — Supplementary Material 1: Supplementary Figures and Tables [file 12933_2024_2156_MOESM1_ESM.docx]

**Supplementary Figure S1. Overview of study participant enrolment**

The individual cause event number refers to the total event experience by the participant in the study. The number of events for individual component may include overlapping individuals who developed more than 1 unique type of event. Abbreviation: AMI, acute myocardial infarction MACE, major adverse cardiovascular events CVD, cardiovascular disease.

**Supplementary Figure S2. Correlation plot between plasma angiogenin measured using Olink and ELISA (N=78 participants: Spearman correlation=0.85)**

**
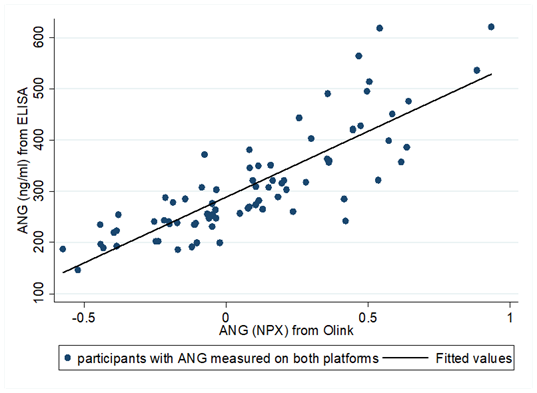
**

**Supplementary Table S1: Baseline clinical and biochemical characteristics in participants included in the current study versus those not included**

|  | Included  (N=1083) | Not included  (N=974) |
| --- | --- | --- |
| Index age (years) | 53.5 ± 10.6 | 61.7 ± 9.3 |
| Male sex (%) | 49.6 | 52.5 |
| Ethnicity (%) |  |  |
| Chinese | 49.4 | 53.2 |
| Malay | 22.7 | 22.3 |
| Asian Indian | 27.9 | 24.5 |
| Diabetes duration (years, IQR) | 5 (3-12) | 12 (8-20) |
| Active smoker (%) | 9.9 | 7.2 |
| ASCVD history (%) | 6.2 | 9.5 |
| Body mass index (kg/m^2^) | 28.3 ± 5.4 | 27.1 ± 5.0 |
| HbA1c (%) | 7.7 ± 1.3 | 7.9 ± 1.4 |
| Blood pressure (mmHg) |  |  |
| Systolic | 137 ± 17 | 146 ± 20 |
| Diastolic | 80 ± 9 | 78 ± 10 |
| Mean arterial pressure | 99 ± 11 | 101 ± 11 |
| Lipid profile (mM) |  |  |
| HDL cholesterol | 1.29 ± 0.36 | 1.29 ± 0.35 |
| LDL cholesterol | 2.81 ± 0.82 | 2.71 ± 0.85 |
| Triacylglycerol (IQR) | 1.35 (1.02-1.88) | 1.48 (1.08-2.03) |
| Baseline renal function |  |  |
| eGFR (ml/min/1.73m^2^) | 98 ± 17 | 70 ± 30 |
| urine ACR (µg/mg, IQR) | 15 (5-49) | 42 (11-296) |
| High sensitivity CRP (ng/ml, IQR) | 2279 (761-5013) | 1858 (549-4382) |
| Medication usage (%) |  |  |
| Aspirin | 17.6 | 29.8 |
| Statin | 77.9 | 84.4 |

Data were presented as mean ± SD, median (interquartile range, IQR) or percentages. Between group differences were compared by student *t* test, Mann-Whitney U test or X^2^ test where appropriate. ASCVD, atherosclerotic cardiovascular disease; eGFR, estimated glomerular filtration function; ACR, albumin-to-creatinine ratio; CRP, c-reactive protein.

**Supplementary Table S2: Correlation coefficients between plasma angiogenin level and clinical variables**

| **Variables** | **Correlation coefficient** |
| --- | --- |
| Age | -0.022 |
| Body mass index | 0.028 |
| Diabetes duration | 0.061^*^ |
| HbA1c | 0.051 |
| Mean arterial pressure | 0.101^**^ |
| HDL-cholesterol | -0.201^***^ |
| LDL-cholesterol | 0.045 |
| Triacylglycerol | 0.173^***^ |
| eGFR | -0.265^***^ |
| Urine ACR | 0.242^***^ |
| High sensitivity CRP | 0.066^*^ |

Correlation of plasma angiogenin level with clinical variables was analysed by non-parametric Spearman bivariate correlation analysis. Nominal p values: ^*^p<0.05, ^**^p <0.01, ^***^p <0.001; eGFR, estimated glomerular filtration rate; ACR, albumin-to-creatinine ratio; CRP, c-reactive protein.

**Supplementary Table S3: Association of plasma angiogenin with acute myocardial infarction, stroke, cardiovascular mortality, and hospitalization due to unstable angina pectoris outcome**

| **Plasma angiogenin** | **Acute myocardial infarction (N=58)** | | **Stroke**  **(N=27)** | | **Cardiovascular death**  **(N=20)** | | **Unstable angina pectoris hospitalization (N=28)** | |
| --- | --- | --- | --- | --- | --- | --- | --- | --- |
|  | **HR (95% CI)** | **P value** | **HR (95% CI)** | **P value** | **HR (95% CI)** | **P value** | **HR (95% CI)** | **P value** |
| **Univariate model** | | | | | | | | |
| **per 1 unit increment *** | **4.02 (2.00-8.09)** | **0.002** | **4.46 (1.63-12.17)** | **0.004** | 2.89 (0.87-9.55) | 0.082 | 2.58 (0.94-7.10) | 0.066 |
| **Multivariable model** | | | | | | | | |
| **per 1 unit increment *** | 2.87 (1.25-6.58) | 0.013 | 2.82 (0.85-9.37) | 0.090 | 0.74 (0.16-3.42) | 0.696 | 2.02 (0.63-6.50) | 0.240 |

Cox proportional hazard regression model: time to components of MACE as outcome.

Multivariable model adjusted for age, sex, ethnicity, CVD history (yes or no), smoking status (current versus others), body mass index, diabetes duration, HbA1c, mean arterial pressure, HDL-cholesterol, LDL-cholesterol, log-transformed triacylglycerol, statin and aspirin usage, baseline eGFR and log-transformed urine ACR.

* One NPX increment is interpreted as doubling of plasma angiogenin concentration measured by proximity extension assay.

| **Plasma angiogenin** | **Unadjusted Model** | | **Multivariable Model 1** | | **Multivariable Model 2** | |
| --- | --- | --- | --- | --- | --- | --- |
|  |  | |  | |  | |
|  | **HR (95% CI)** | **P value** | **HR (95% CI)** | **P value** | **HR (95% CI)** | **P value** |
| **Continuous variable**  **(per 1 unit increment) *** | 1.86 (0.87-3.94) | 0.107 | 1.16 (0.50-2.69) | 0.726 | 0.69 (0.28-1.69) | 0.422 |
| **Categorical variable** | | | | | | |
| **Lower tertile** | reference | | reference | | reference | |
| **Intermediate tertile** | 1.50 (0.67-3.33) | 0.324 | 1.27 (0.56-2.86) | 0.567 | 1.06 (0.46-2.41) | 0.900 |
| **Upper tertile** | **2.84 (1.38-5.85)** | **0.005** | 1.98 (0.92-4.26) | 0.080 | 1.37 (0.61-3.08) | 0.449 |

**Supplementary Table S4: Association of plasma angiogenin with all-cause mortality**

Cox proportional hazard regression model: time to all-cause mortality as outcome.

Model 1 adjusted for age, sex, ethnicity, CVD history (yes or no), smoking status (current versus others), body mass index, diabetes duration, HbA1c, mean arterial pressure, HDL-cholesterol, LDL-cholesterol, log-transformed triacylglycerol, statin and aspirin usage.

Model 2 additionally adjusted for baseline eGFR and log-transformed urine ACR above model 1.

* One NPX increment is interpreted as doubling of plasma angiogenin concentration measured by proximity extension assay.

**Supplementary Table S5: Association of plasma angiogenin with MACE (patients with eGFR > 90ml/min/1.73m^2^) (N=776)**

| **Plasma angiogenin**  **(Event number=61)** | **Unadjusted Model** | | **Multivariable Model 1** | | **Multivariable Model 2** | |
| --- | --- | --- | --- | --- | --- | --- |
|  | **HR (95% CI)** | **P value** | **HR (95% CI)** | **P value** | **HR (95% CI)** | **P value** |
| **Continuous variable**  **(per 1 unit increment) *** | **3.03 (1.50-6.14)** | **0.002** | **2.73 (1.22-6.11)** | **0.015** | **2.35 (1.01-5.43)** | **0.047** |
| **Categorical variable** | | | | | | |
| **Lower tertile** | reference | | reference | | reference | |
| **Intermediate tertile** | 1.57 (0.81-3.04) | 0.184 | 1.48 (0.75-2.95) | 0.261 | 1.36 (0.68-2.72) | 0.389 |
| **Upper tertile** | **2.57 (1.35-4.87)** | **0.004** | 1.96 (0.96-4.02) | 0.065 | 1.69 (0.81-3.53) | 0.162 |

Cox proportional hazard regression model: time to 4-point MACE as outcome.

Model 1 adjusted for age, sex, ethnicity, CVD history (yes or no), smoking status (current versus others), body mass index, diabetes duration, HbA1c, mean arterial pressure, HDL-cholesterol, LDL-cholesterol, log-transformed triacylglycerol, statin and aspirin usage.

Model 2 additionally adjusted for baseline eGFR and log-transformed urine ACR above model 1.

* One NPX increment is interpreted as doubling of plasma angiogenin concentration measured by proximity extension assay.

**Supplementary Table S6: Association of plasma angiogenin with MACE in participants stratified by ASCVD history, albuminuria and HbA1c category**

| **Plasma angiogenin**  **(one NPX unit)** | **Unadjusted Model** | **P value** | **Multivariable Model** | **P value** | **P value (Interaction)** |
| --- | --- | --- | --- | --- | --- |
| *ASCVD history* | | | | | 0.950 |
| **With ASCVD history** | 2.71 (0.69-10.7) | 0.155 | 27.7 (1.97-391) | 0.014 |  |
| N=66 |  |  |  |  |  |
| Event number = 18 |  |  |  |  |  |
| **Without ASCVD history** | 2.89 (1.63-5.14) | <0.001 | 1.50 (0.75-2.99) | 0.254 |  |
| N=993 |  |  |  |  |  |
| Event number = 86 |  |  |  |  |  |
|  | | | | | |
| *Albuminuria category* | | | | | 0.560 |
| **Non-albuminuria (ACR <30mg/g)** | 2.55 (1.19-5.50) | 0.017 | 1.74 (0.73-4.15) | 0.215 |  |
| N = 685 |  |  |  |  |  |
| Event number = 55 |  |  |  |  |  |
| **Albuminuria (ACR ≥30 mg/g)** | 2.98 (1.43-6.19) | 0.004 | 2.16 (0.91-5.12) | 0.080 |  |
| N = 374 |  |  |  |  |  |
| Event number = 49 |  |  |  |  |  |
|  |  |  |  |  |  |
| *HbA1c* | | | | | 0.404 |
| **HbA1c <8%** | 3.20 (1.52-6.73) | 0.002 | 2.61 (1.08-6.30) | 0.032 |  |
| N = 686 |  |  |  |  |  |
| Event number = 54 |  |  |  |  |  |
| **HbA1c ≥8%** | 2.60 (1.26-5.38) | 0.010 | 1.66 (0.65-4.25) | 0.287 |  |
| N = 373 |  |  |  |  |  |
| Event number = 50 |  |  |  |  |  |

Cox proportional hazard regression model: time to MACE as outcome.

Multivariable model adjusted for age, sex, ethnicity, CVD history (yes or no), smoking status (current versus others), body mass index, diabetes duration, HbA1c, mean arterial pressure, HDL-cholesterol, LDL-cholesterol, log-transformed triacylglycerol, statin and aspirin usage, baseline eGFR and log-transformed urine ACR; ASCVD, atherosclerotic cardiovascular disease; ACR, albumin-to-creatinine ratio; CRP, c-reactive protein; N, total number of patient in the sub-group.
